# Parent-mediated play‐based interventions to improve social communication and language skills of preschool autistic children: A systematic review and meta-analysis protocol

**DOI:** 10.1371/journal.pone.0270153

**Published:** 2022-08-15

**Authors:** Emre Deniz, Gill Francis, Carole Torgerson, Umar Toseeb

**Affiliations:** Department of Education, University of York, York, United Kingdom; Radboud University: Radboud Universiteit, NETHERLANDS

## Abstract

Early years interventions have shown to be effective in improving the social communication and language skills of autistic children. Therefore, various play-based interventions have been developed to support those developmental areas of autistic children. Although researchers have previously reported the overall effectiveness of different types of play-based interventions on the social communication and language skills of autistic children, no previous systematic reviews have yet evaluated the effectiveness of parent-mediated play-based interventions in preschool autistic children. The overarching aims of the study will be to (i) report the key characteristics and (ii) synthesise the results of studies evaluating parent-mediated play-based interventions targeting the social communication and language skills of preschool autistic children using experimental designs. A comprehensive search for and screening of the relevant studies published between 2000 and 2021 will be undertaken. To be included, studies will have to (i) use either a randomised control trial or quasi-experimental design, (ii) focus on preschool autistic children aged six years old or younger, (iii) deliver a play-based intervention in non-educational settings, and (iv) include at least one parent as the mediator of the intervention. Data extraction of all included studies will be undertaken using a specially devised template and they will also be assessed for risk of bias using an adapted form from the Cochrane Risk of Bias tool. The overall characteristics of the included studies will be reported and a narrative synthesis of the results of the included studies will be undertaken. A meta-analysis may be performed (if justified) to report the pooled effect size of the parent-mediated play-based interventions on the social communication and language skills of preschool autistic children.

**Trial registration:** The current study protocol was pre-registered with the international prospective register of systematic reviews (PROSPERO: CRD42022302220).

## Introduction

Although it is notoriously difficult to define the nature of play, as it is a wide-ranging term and intuitive understandings differ across disciplines, researchers generally agree on its following characteristics: (i) not obligatory or serious, (ii) ungoverned by external rules, (iii) free and spontaneous, (iv) intrinsically motivated, (v) pleasurable [1–5]. Based on the aforementioned characteristics, the current study is guided by Rubin et al.’s [6] definition of play as activities that are intrinsically motivated, free to choose, spontaneous, purified from externally imposed rules, self-guided, non-literal, and require active engagement.

Play is an indispensable activity in children’s lives as it contributes to many areas of child development including cognitive, social communication, language, emotional, and physical development. It has been suggested that symbolic play is effective in supporting perspective-taking [7], creativity [8], representational understanding [9], and theory of mind skills [10]. Play is instrumental in supporting children’s various social communication skills such as joint attention and functional play [11], joint engagement [12, 13], social interaction [14], and social competence [15]. Regarding language skills, play has been found to improve the overall language [16] as well as expressive and receptive language skills [17, 18], the total words children produce, mean length of utterance, and linguistic complexity and diversity in at-risk preschool children [19]. Play is widely used as a therapeutic tool [20, 21] and has been found to have a significant role in decreasing children’s internalising [22] and externalising problems [23, 24] and contributing to their healthy emotional development [25, 26]. Lastly, play provides children with limitless opportunities to be physically active in daily life, thus, helps them to develop muscles and gain physical strength [27].

Autism is a pervasive neurodevelopmental condition with a prevalence of approximately 1 in 44 children in the US [28]. Autism is characterised by persistent deficits in social communication, and language skills [29]. Autistic individuals may exhibit some or all of the following: deficits in social-emotional reciprocity, reduced sharing of interests, failure to initiate or respond to social interactions, deficits in nonverbal (e.g., eye contact, gestures, and facial expressions), and verbal communication. In addition, research have shown that autistic children have reduced levels of social communication [30–32], and language [33] abilities compared to their neurotypical peers. However, there is considerable heterogeneity as affected individuals have varied profiles of strengths and difficulties.

Many play-based interventions have been developed to support the social communication and language skills of autistic children [34, 35]. For instance, the Joint Attention Symbolic Play Engagement and Regulation (JASPER) intervention has repeatedly been found to be effective in improving joint attention, joint engagement, child-parent interaction, symbolic play, and language skills of autistic children [36–39]. Additionally, Pivotal Response Treatment (PRT) is effective in improving the social communication and language skills of autistic children [40–42]. Similarly, Child-Centred Play Therapy (CCPT) has also been found to be a significant intervention in increasing social participation and reciprocity skills and decreasing detrimental social behaviours [43–45]. Although different types of play-based interventions have been shown to be effective, the characteristics of such interventions vary mainly based on their primary focus, type of play, implementers, and mediators.

While the majority of play-based interventions have solely been built on the interaction between a child and interventionist, there has been a recent trend in the inclusion of mediators such as peers, teachers, and parents. This, perhaps, is not only due to the mediators’ ability and availability in mediating such interventions but also targeting an improvement in both children’s and mediators’ outcomes within one intervention [46]. Unlike parent-mediated interventions, peer- and teacher-mediated interventions are often based on group play activities, conducted in educational settings, and are built on the elements of role-play and social reinforcement [47, 48]. Both peer and teacher-mediated interventions have been reported to be significant in improving social communication and language skills [49–51], as well as core autistic traits [52].

Parent-mediated interventions, however, differ from peer- and teacher-mediated interventions in the sense that they are often one-to-one activities between a parent and a child, take place in non-educational settings (e.g., home, clinics), and are commonly based on the principles of Mediated Learning Theory [53]. Parent-mediated interventions have been found to have significant positive effects on certain developmental skills of autistic children such as joint attention, social interaction, language, nonverbal communication, and adaptive functioning skills [54]. Additionally, a recent meta-analysis has reported a significant effect size for parent-mediated interventions on the communication and language skills of preschool autistic children [55]. Similarly, some researchers have found that parent-mediated interventions are effective in increasing language comprehension and child-parent interaction, as well as improving autistic traits in young autistic children [56].

Regarding the effectiveness of *parent-mediated play-based interventions*, JASPER has been found effective in increasing the joint engagement and play skills of preschool autistic children [46]. Additionally, the Play and Language for Autistic Youngsters (PLAY) intervention significantly improved social and emotional functioning and reduced autism severity in preschool autistic children [57]. In another study, a one-year Focused Playtime Intervention (FPI) has been reported to have significant positive effects on the expressive language skills of preschool autistic children [58]. A pilot study has reported that a parent-mediated Developmental, Individual-differences, Relationship-based model (DIR/Floortime) intervention significantly improved the functional and emotional skills and core autistic traits of preschool autistic children [59]. Although individual studies reported the effectiveness of parent-mediated play-based interventions, to the best of the authors’ knowledge, no previous systematic review or meta-analysis has reported the characteristics and effectiveness of parent-mediated play-based interventions in preschool autistic children. Thus, the characteristics and overall effectiveness of such interventions remain unknown.

In the current protocol, the following elements of the proposed systematic review and meta-analysis are reported: (i) aims, (ii) research questions, (iii) study methodology (e.g., target sample, search strategy, inclusion criteria, predictor and outcome variables, data extraction, quality appraisal, and data analysis), and (iv) conflict of interest. The overarching aims of the study will be to (i) report the key characteristics and (ii) synthesise the results of studies evaluating parent-mediated play-based interventions targeting the social communication and language skills of preschool autistic children using experimental designs. To address these aims, the following research questions will be asked:

- What are the key characteristics of parent-mediated play-based interventions?
- How effective are parent-mediated play-based interventions in improving the social communication and language skills of preschool autistic children?
- What factors, if any, mediate/moderate the impact of parent-mediated play-based interventions on children’s social communication and language outcomes?

## Methods

### Study protocol and registration

The current study protocol was developed by following the Preferred Reporting Items for Systematic Review and Meta-Analysis Protocols (PRISMA-P) [60] checklist (S1 Table in S1 File) and pre-registered with the international prospective register of systematic reviews (PROSPERO) network to improve transparency, reduce potential bias and prevent duplication of another study (Ref: CRD42022302220). The results and supporting documents of the current study will be made publicly available on the open science framework and submitted to a peer-reviewed journal for publication upon completion of the study.

### Study design

The current review will focus on the studies that applied randomised controlled trials or quasi-experimental designs with at least one control group or condition with the following characteristics: (i) a non-treated control group, with or without waitlist design, (ii) a control group that received treatment other than a play-based intervention.

### Target sample

The current review aims to search for and screen for studies that focus on preschool autistic children with social communication and language needs who were aged six years old or younger when the research was undertaken. Studies with a sample of children with a previously defined form of autism, such as Asperger’s syndrome, autistic spectrum disorder, and pervasive developmental disorder-not otherwise specified (based on older versions of the DSM-V), will be included in this review.

### Intervention characteristics

The current review will include evaluations of parent-mediated play-based interventions that were conducted in non-educational (e.g., home, clinics, etc) settings and targeted social communication and language needs of preschool autistic children. Studies that did not report any information regarding the intervention setting will be treated as conducted in *a non-educational setting* and included in the sample as long as they were not delivered or mediated by teachers. Additionally, taking Rubin et al. ’s [6] play approach into account, interventions that were not solely based on play, delivered during the playtime or within the playground will be excluded from this review. The latter criterion will be met in the following conditions:

- The intervention is a known and named play-based intervention (e.g., JASPER, PLAY, etc).
- The intervention is a known and named therapeutic approach that uses play as a therapeutic tool (e.g., play therapy, child-centred play therapy, cognitive behavioural play therapy, Rogerian play, etc).
- The intervention is a known and named developmental intervention that is built on the elements of play or consists of play-based activities such as joint attention, joint engagement, parent-child play (DIR Floortime, Pivotal Response Treatment, Early Start Denver Model, etc).
- The intervention is not a previously validated and named intervention but is judged as a play-based intervention by the authors based on the characteristics of the intervention.

The last criterion will be met if “play is explicitly mentioned” [61] or if the intervention consists of parent-child play or child-led play activities. That is, any interventions that are not solely based on play are judged as *not play-based* (behavioural modification techniques, teaching and training activities, gaming, etc) will be excluded from the sample. Such interventions may include but are not limited to the following: Video modelling, video prompting, activity schedules, script fading, behavioural interventions, music therapy, virtual reality, gaming, and computer/video games, tablet applications.

### Predictor and outcome variables

The predictor variables will be parent-mediated play-based interventions that targeted social communication and language outcomes of preschool autistic children. The included outcome variables of the current study will be broadly set as all variables that are related to children’s social communication (e.g., child-parent interactions, child-peer interactions, joint attention, joint engagement, joint interest, play skills, eye contact, social responsiveness, positive social interaction, initiation of social interaction, child’s social functioning, etc), language skills (e.g., expressive and receptive language skills, vocabulary, the number of spoken words, the mean length of utterance, etc). Additionally, autistic traits will also be included in the outcome variables if one or more studies reported autistic traits (e.g., restricted repetitive behaviours, social affect, calibrated severity score, etc) as primary outcome variables.

### Study inclusion and exclusion criteria

The following criteria were pre-defined for the inclusion of the studies during the title and abstract screening phases [62]:

- Studies targeting children and youth younger than 19 years of age.
- Studies with a sample of autistic children, including the ones who screened as "high likelihood for autism" on a validated autism screening measure, or children with a previously diagnosed form of autism (e.g., Asperger’s syndrome, PDD-NOS, autistic spectrum disorder).
- Studies with randomised controlled trial (RCT) or quasi-experimental (QE) designs.
- Studies that delivered a play-based intervention.
- Studies that are primarily focused on social communication, and language outcomes.
- Studies published between 2000 and 2021, including grey literature.
- Studies that are published in English.

The predefined criteria were previously applied in Francis et al.’s [62] review which undertook an overarching in-depth review of children with social communication and language needs but reported only on mental health outcomes. The current review is undertaken by the same team and uses the same predefined criteria to study social communication and language outcomes using the same data identified from the database screening phase described in Francis et al. The inclusion criteria will be further narrowed at the full-text screening stage based on the focus of this review to select:

- Studies targeting preschool autistic children aged six years old (72 months) or younger.
- Studies including at least one parent as a mediator. Studies with multiple implementers will also be included if implementers are placed in separate intervention groups alongside a non-play control group (e.g., intervention group 1 = parent-mediated intervention, intervention group 2 = sibling-mediated intervention, and a control group).
- Studies that were conducted in non-educational settings (i.e., home, nursery, clinic, etc).

Additionally, the following exclusion criteria will be applied at the full-text screening stage:

- Studies will be excluded if the upper age limit of their sample was greater than six years old (72 months) unless the authors separately reported the outcomes of those aged six years old or younger.
- Studies will be excluded if the participants in the sample were not formally diagnosed with or screened for autism.
- Pre-experimental studies with no control groups will be excluded.
- Studies that compared the effectiveness of two or more play-based interventions will be excluded unless they provide a control group that received a non-play-based intervention,
- Studies that delivered a play-based intervention that does not meet the concept of play, that is defined in the current review, will be excluded.
- Studies will be excluded if there are multiple implementers in a single intervention group (e.g., intervention group = parent and sibling mediated play intervention and a control group).

### Study selection procedure

**Fig 1:**
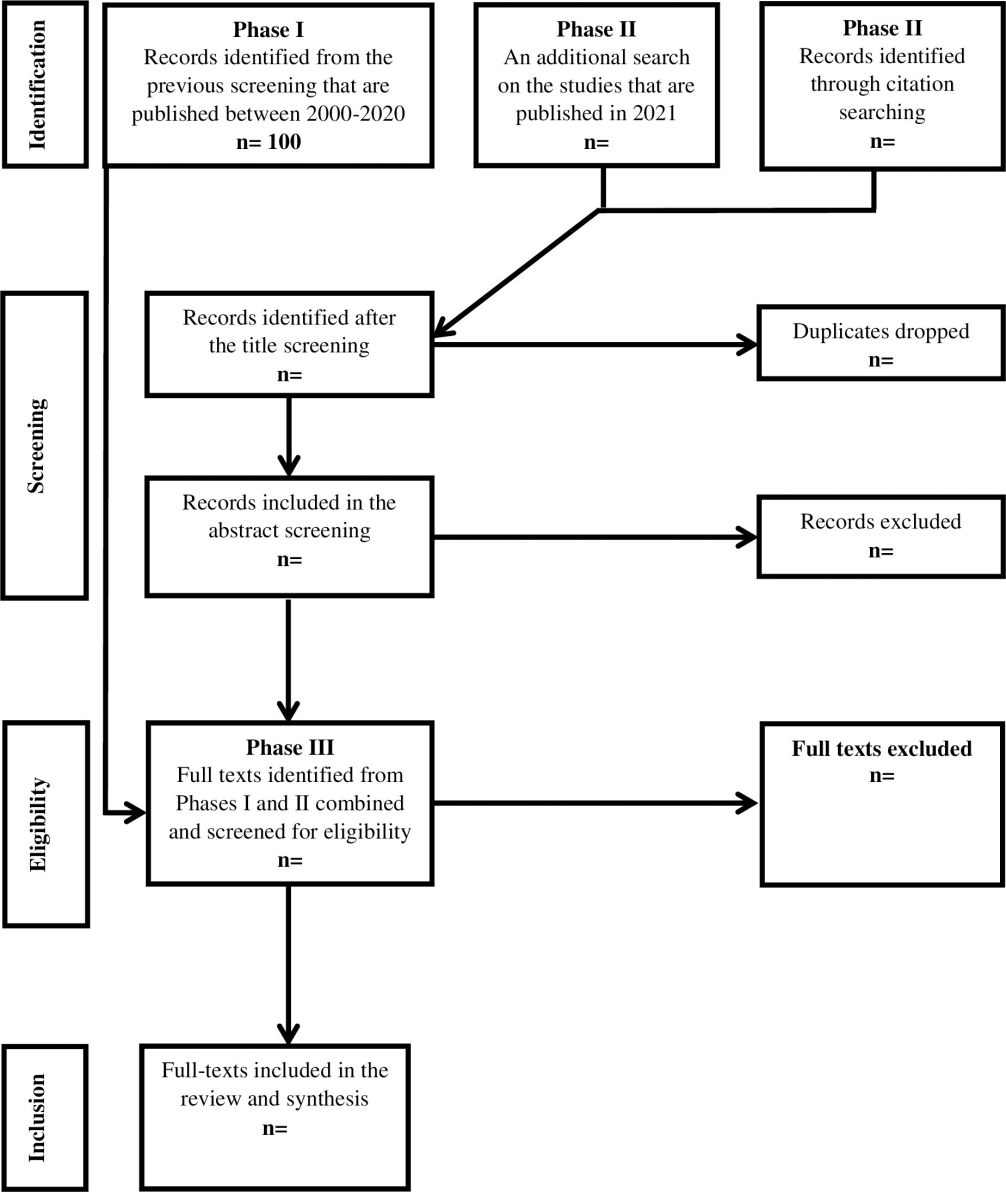
Prisma flow diagram for records of literature screening.

As mentioned previously, the current study is part of an overarching review [62]. In the overarching review, 110 studies that reported the effectiveness of play-based interventions on the social communication and language skills or mental health of children with social communication and language needs were found after the full-text screening. The previous in-depth review reported on the (n = 10) studies that targeted mental health outcomes. The remaining studies (n = 100) are the starting point for identifying relevant studies for this in-depth review on the effectiveness of play-based parent-mediated interventions on *social communication* and *language* outcomes. Additional searching and screening will be carried out to capture recently published studies post the completion of Francis, et al. The current review, therefore, comprises three screening phases, outlined in Fig 1.

#### Phase I: The overarching review

In phase I, the authors carried out an overarching review, guided by Cochrane [63] and PRISMA-P [60]. Searching was undertaken on the following databases: Ebscohost (including ERIC), Ethos, ProQuest, PsycINFO, Pubmed, Scopus, Web of Science, and Google Scholar. The following predefined search strings were used to capture the studies that met the inclusion criteria: “Play”, “speech”, “language”, “communication” "autism", “autistic”, and “Asperger’s” and named play interventions (e.g., JASPER, PRT, CCPT). In addition to the master terms, Boolean search strategies were also adopted by combining those pre-specified master keywords with some useful operators such as “AND”, “OR”, “NOT”. and “quotation marks’’.

The database screening was conducted by one author (ED) using the search strings which resulted in 1785 potentially relevant studies after duplicates were dropped. Title and abstract screening was then carried out by two authors (ED & GF), independently. The two authors agreed on the inclusion of n = 366 studies, with a high inter-coder agreement (κ = .88 (95% CI, .85- .91). Disagreements were discussed and resolved within the research team. Lastly, a double screening of the full texts, by the same authors, indicated n = 100 studies addressing social communication and language outcomes and, within those 100 studies, n = 10 addressing mental health outcomes. The studies addressing social communication and language outcomes are the focus of this in-depth systematic review. In the current study, therefore, the pre-identified studies addressing social communication and language outcomes will be included and rescreened according to the relevant inclusion criteria. More information regarding the screening for Phase I is reported in the previous review [62].

#### Phase II: Update

Since Phase I comprises the studies that were published between 2000 and January 2021, an additional search for relevant studies published in 2021 will be carried out on the aforementioned databases and by using the same search strings (S2 Table in S1 File). The database search will be conducted by the first author (ED) alongside a comprehensive citation search of the previous systematic review and meta-analysis on parent-mediated interventions in autism [39, 51, 54–56, 64–73]. The identified studies will be merged and downloaded into Mendeley Reference Manager software for performing the title, abstract and full-text screenings. The title and abstract screenings will then be conducted by two authors, (ED & GF), to make sure no eligible study is overlooked. Any discrepancies between the two coders, at the title and abstract screening, will be resolved by the third author’s opinion (CT). Lastly, the full-text screening will be carried out by two authors (ED & CT), independently, and disagreements will be resolved by the inclusion of a third reviewer (GF), prior to the final decision.

#### Phase III: Current in-depth review

In Phase III, firstly, the results from Phase I and Phase II will be merged, and duplicate studies will be dropped. Secondly, all studies will be rescreened by the four authors according to three additional eligibility criteria for this in-depth SR: (i) studies focusing on preschool autistic children aged six years old or younger, (ii) interventions that were conducted in non-educational settings, (iii) interventions that include at least one parent as a mediator. Eligible studies will then be included in the review and carried over to the data extraction stage.

### Data extraction

#### Descriptive data

Information related to intervention types, play approaches, sample sizes, experimental designs, etc, will be extracted to report the included study characteristics.

#### Quantified outcomes

Quantified results related to the social communication and language skills of autistic children will be extracted from the included studies for the narrative synthesis and meta-analysis (if justified). The authors will independently double data extract all quantified (primary and secondary) outcomes (not exploratory outcomes). All outcome measures will be reported. Should a meta-analysis be deemed appropriate, the authors will extract one outcome for each domain: social communication, language, and autistic traits. We will report one outcome for each domain using the following pre-specified algorithm: the pre-specified primary outcome(s) (where provided); the first outcome reported in text or table in the paper; if the first outcome is a subscale measure, the authors will use the total score of the sub-scale instead. Quantified outcomes will include numbers, means, standard deviations, standard errors, confidence intervals, and reported effect sizes that are reported regarding the effects of the delivered intervention on the social communication and language skills as well as autistic traits of preschool autistic children.

All data will be double extracted using a specially devised data extraction form (S3 Table in S1 File). The first author will extract the data from all included studies and each of the other authors (GF, CT, & UT) will extract data from a third of the sample studies. Any discrepancy between the coders regarding the extracted data will be discussed and resolved. If two coders are not able to come to an agreement, the third and fourth coders will be consulted. Upon completion of the data extraction, all numerical data will be extracted by using the quantified outcomes form (S4 Table in S1 File).

### Quality appraisal

The methodological quality of the included studies will be assessed by all four authors with the first author assessing all (ED) and each of the others (CT, GF, & UT) assessing a third of the sample, independently. The authors will be using two risk of bias (RoB) tools one of which is for randomised control trials and the other for quasi-experimental designs (S5 Table in S1 File). Both RoB forms were previously adapted [62] from the revised Cochrane Risk of Bias Tool for Randomized Trials (RoB 2) [74]. and Cochrane Risk of Bias Tool in Non-randomized Studies of Interventions’ (ROBINS-1) [75]. Each tool has five domains that assess the following items: random allocation and concealment or allocation, blinding to the intervention, appropriate measurement, reported and selected outcomes, a prior analysis plan and a protocol.

Studies will be categorised under three categories: (i) low risk of bias, (ii) some concerns of bias, and (iii) high risk of bias by summarising the scores from five domains. The overall RoB will be judged as *low risk of bias* if the study is judged to be at low risk of bias for all domains, *some concerns* if any domains are judged as some concerns, and *high* if any domains are judged as high risk of bias [74]. Although the level of risk of bias will not result in the exclusion of any studies from the review’s sample, studies with a high risk of bias will be excluded from the meta-analysis (if undertaken). Furthermore, sensitivity analysis may be conducted to assess whether the methodological quality of studies (e.g., low risk of bias, some concerns) showed any impact on the reported effect size of the interventions. Additional subgroup sensitivity analyses may also be used to check if intervention effects vary by research design (RCT or QED) if there are sufficient relevant studies.

### Data analysis and synthesis

All statistical analyses will be conducted using statistical software R. If the interventions and outcomes are judged to be sufficiently homogeneous, a meta-analysis may be performed to report the overall effect sizes of the play-based interventions on the social communication and language skills of preschool autistic children with social communication and language needs. To do this, the reported outcome variables will be categorised under three umbrella terms: (i) social communication skills, (ii) language skills, and (iii) autistic traits. Also, an overall effect size may be reported by calculating the standardised mean difference (*Cohen’s d*) on all reported social communication, language, and autism severity outcomes. Confidence intervals (CI, 95%) and standard errors (SE) will also be reported alongside the reported effect sizes. Additionally, statistical heterogeneity may be assessed using either *Cochran’s Q (x^2^)* or *I^2^* depending on the sample size of the meta-analysis. Given that the data on study outcomes, in this context, is likely to be heterogeneous, a random-effects analysis may be conducted to report the degree of variations between the study outcomes.

### Missing data

All included studies will be checked in terms of missing data. In case any missing data is found, the research team will discuss whether the missing part of the data affects the reported outcomes, if so, three solutions may be applied to deal with the missing data. First, the research team will try to contact the studies’ corresponding authors to ask if the missing data could be shared. Second, the research team will try to impute the missing data by using other-reported quantified outcomes. For instance, in cases where the standard deviations (SD) are not provided, the research team will use the mean and sample size to calculate the SDs. Third, if corresponding authors do not respond and the reported information in the study is insufficient to impute the missing data, the study will be excluded from the meta-analysis, if undertaken.

## Discussion

Play has a significant role in contributing to children’s development [76]. Therefore, various play-based interventions have been developed and used to support the social communication and language needs of autistic children [11–19]. Although many reviews and meta-analyses have reported the characteristics and overall effectiveness of play-based interventions on autistic children’s social communication and language skills [39, 51, 54–56, 64–73], to the best of the authors’ knowledge, the current study will be the first to evaluate the effectiveness of parent-mediated play-based interventions conducted in non-educational settings on the social communication and language skills of preschool autistic children. Hence, the review will shed new light on the role of parental mediation in the effectiveness of play-based interventions.

It is anticipated that the current study will have many strengths and some potential limitations. A major strength is that having a pre-registered study protocol that comprises an apriori analysis plan will minimise any potential for bias in study selection and identification of outcomes. In addition, the design, conduct and reporting of the current study will be guided by the PRISMA statement and an adapted version of the widely used Cochrane quality assurance tools to evaluate the risk of bias in the included studies. Since autism is highly heritable [77, 78], parent-mediated interventions are likely to contribute to parental outcomes alongside child-level outcomes [66, 79]. However, the research questions for the review focus on child-level outcomes only (social communication and language). This means that parental outcomes are not relevant to the RQs and so have not been included. Future systematic reviews could focus on research questions that address the effectiveness of parent-mediated play-based interventions on parental outcomes as these are valid and significant RQs.

## Supporting information

S1 File PRISMA-P checklist, search strings, data extraction form, quantified outcomes, and risk of bias assessment tool. (DOC)

## References

[1] Burghardt GM. The comparative reach of play and brain: perspective, evidence, and implications. American Journal of Play. 2010;2(3):338–56.

[2] Eberle SG. The elements of play: Toward a philosophy and a definition of play. American Journal of Play. 2014;6(2):214–33.

[3] Hughes FP. Spontaneous play in the 21st century. Contemporary perspectives on play in early childhood education. IAP, 2003 Feb 1:21–39.

[4] Huizinga J. Homo Ludens: A study of the play-element in our culture. Routledge & Kegan Paul; 1949.

[5] Saracho ON, Spodek B. A historical overview of theories of play. Multiple perspectives on play in early childhood education. New York: State University of New York Press, 1998 Jan 15:1–0.

[6] Rubin KH, Fein GG, Vandenberg B. Play In Mussen PH & Hetherington EM (Eds.), Handbook of child psychology vol. 4: socialization, personality, and social development (Vol. 4, pp. 693–774). New York; Chichester: Wiley, 1983.

[7] Bergen D. The role of pretend play in children’s cognitive development. Early Childhood Research & Practice. 2002;4(1):n1.

[8] Saracho O. Young children’s creativity and pretend play. Early Child Development and Care. 2002 Oct 1;172(5):431–8. 10.1080/03004430214553

[9] Vygotsky LS. Play and its role in the mental development of the child. Undefined. 1967 Apr;5(3):6–18.

[10] Lillard AS, Kavanaugh RD. The contribution of symbolic skills to the development of an explicit theory of mind. Child Development. 2014 Jul;85(4):1535–51. doi: 10.1111/cdev.12227 24502297

[11] So WC, Cheng CH, Lam WY, Huang Y, Ng KC, Tung HC, et al. A robot-based play-drama intervention may improve the joint attention and functional play behaviors of chinese-speaking preschoolers with autism spectrum disorder: a pilot study. Journal of Autism and Developmental Disorders. 2020 Feb;50(2):467–81. doi: 10.1007/s10803-019-04270-z 31655965

[12] Goods KS, Ishijima E, Chang YC, Kasari C. Preschool based JASPER intervention in minimally verbal children with autism: Pilot RCT. Journal of Autism and Developmental Disorders. 2013 May;43(5):1050–6. doi: 10.1007/s10803-012-1644-3 22965298 PMC4222903

[13] Shire SY, Gulsrud A, Kasari C. Increasing responsive parent–child interactions and joint engagement: Comparing the influence of parent-mediated intervention and parent psychoeducation. Journal of Autism and Developmental Disorders. 2016 May;46(5):1737–47. doi: 10.1007/s10803-016-2702-z 26797940 PMC4826805

[14] Hu X, Zheng Q, Lee GT. Using peer-mediated LEGO® play intervention to improve social interactions for Chinese children with autism in an inclusive setting. Journal of Autism and Developmental Disorders. 2018 Jul;48(7):2444–57. doi: 10.1007/s10803-018-3502-4 29453705

[15] Stagnitti K, O’Connor C, Sheppard L. Impact of the learn to play program on play, social competence and language for children aged 5–8 years who attend a specialist school. Australian Occupational Therapy Journal. 2012 Aug;59(4):302–11. doi: 10.1111/j.1440-1630.2012.01018.x 22934903

[16] O’Connor C. and Stagnitti K., 2011. Play, behaviour, language and social skills: The comparison of a play and a non-play intervention within a specialist school setting. Research in Developmental Disabilities, 32(3), pp.1205–1211. doi: 10.1016/j.ridd.2010.12.037 21282038

[17] Danger S, Landreth G. Child-centered group play therapy with children with speech difficulties. International Journal of Play Therapy. 2005;14(1):81. https://psycnet.apa.org/doi/10.1037/h0088897

[18] Han M, Moore N, Vukelich C, Buell M. Does play make a difference? How play intervention affects the vocabulary learning of at-risk preschoolers. American Journal of Play. 2010;3(1):82–105.

[19] Craig-Unkefer LA, Kaiser AP. Improving the social communication skills of at-risk preschool children in a play context. Topics in Early Childhood Special Education. 2002 Jan;22(1):3–13. https://doi.org/10.1177%2F027112140202200101

[20] Axline VM. Play Therapy, The inner dynamics of childhood. Cambridge: Houghton Mifflin. 1947.

[21] Knell SM. Cognitive-behavioral play therapy. Northvale, N.J.; London: Jason Aronson;1995.

[22] Shahriari Y, Ghasemzadeh S, Vakili S. The effectiveness of child-centred play therapy on internalization and extrapolation behavioral problems in children with cerebral palsy. Iranian Journal of Psychiatry and Clinical Psychology. 2019 Sep 10;25(3):236–49.

[23] Meany‐Walen KK, Kottman T, Bullis Q, Dillman Taylor D. Effects of adlerian play therapy on children’s externalizing behavior. Journal of Counseling & Development. 2015 Oct;93(4):418–28. 10.1002/jcad.12040

[24] Ritzi RM, Ray DC, Schumann BR. Intensive short-term child-centered play therapy and externalizing behaviors in children. International Journal of Play Therapy. 2017 Jan;26(1):33. https://psycnet.apa.org/doi/10.1037/pla0000035

[25] Woolf A. Everyone playing in class: A group play provision for enhancing the emotional well‐being of children in school. British Journal of Special Education. 2011 Dec;38(4):178–90. 10.1111/j.1467-8578.2011.00520.x

[26] Lee RL, Lane S, Brown G, Leung C, Kwok SW, Chan SW. Systematic review of the impact of unstructured play interventions to improve young children’s physical, social, and emotional wellbeing. Nursing & Health Sciences. 2020 Jun;22(2):184–96. 10.1111/nhs.12732 32358875

[27] Anderson-McNamee JK, Bailey SJ. The importance of play in early childhood development. Montana State University Extention. 2010;4(10):1–4.

[28] Centers for Disease Control and Prevention. Data & statistics on autism spectrum disorder [internet]. 2022. [updated 2022 March 2; cited 2022 April 27]. Available from: https://www.cdc.gov/ncbddd/autism/data.html.

[29] American Psychiatric Association. Diagnostic and statistical manual of mental disorders: DSM-5. Washington, DC: American psychiatric association; 2013 May.

[30] Bauminger N, Shulman C, Agam G. Peer interaction and loneliness in high-functioning children with autism. Journal of Autism and Developmental Disorders. 2003;33(5):489–507. doi: 10.1023/a:1025827427901 14594329

[31] Volkmar FR, Lord C, Bailey A, Schultz RT, Klin A. Autism and pervasive developmental disorders. Journal of Child Psychology and Psychiatry. 2004;45(1):135–70. doi: 10.1046/j.0021-9630.2003.00317.x 14959806

[32] Tager‐Flusberg H, Joseph R, Folstein S. Current directions in research on autism. Mental Retardation and Developmental Disabilities Research Reviews. 2001;7(1):21–9. 10.1002/1098-2779(200102)7:1<21::AID-MRDD1004>3.0.CO;2-3 11241879

[33] Tager-Flusberg H, Rogers S, Cooper J, Landa R, Lord C, Paul R, et al. Defining spoken language benchmarks and selecting measures of expressive language development for young children with autism spectrum disorders. Journal of Speech, Language, and Hearing Research. 2009 Jun 1;52(3):643–53. 10.1044/1092-4388(2009/08-0136) PMC2819321 19380608

[34] Pasco G. The value of early intervention for children with autism. Paediatrics and Child Health. 2018 Aug 1;28(8):364–7. 10.1016/j.paed.2018.06.001

[35] Zwaigenbaum L, Bauman ML, Choueiri R, Kasari C, Carter A, Granpeesheh D, et al. Early intervention for children with autism spectrum disorder under 3 years of age: recommendations for practice and research. Pediatrics. 2015;136(Supplement_1): S60–81. doi: 10.1542/peds.2014-3667E 26430170 PMC9923898

[36] Gulsrud AC, Hellemann G, Shire S, Kasari C. Isolating active ingredients in a parent-mediated social communication intervention for toddlers with autism spectrum disorder. Journal of Child Psychology and Psychiatry. 2016 May;57(5):606–13. doi: 10.1111/jcpp.12481 26525461 PMC8320675

[37] Kasari C, Freeman S, Paparella T. Joint attention and symbolic play in young children with autism: A randomized controlled intervention study. Journal of Child Psychology and Psychiatry. 2006 Jun;47(6):611–20. doi: 10.1111/j.1469-7610.2005.01567.x 16712638

[38] Shire SY, Shih W, Chang YC, Bracaglia S, Kodjoe M, Kasari C. Sustained community implementation of JASPER intervention with toddlers with autism. Journal of Autism and Developmental Disorders. 2019 May;49(5):1863–75. doi: 10.1007/s10803-018-03875-0 30627891

[39] Waddington H., Reynolds J. E., Macaskill E., Curtis S., Taylor L J., & Whitehouse, A. J. (2021). The effects of JASPER intervention for children with autism spectrum disorder: A.systematic review. Autism, 25(8), 2370–2385. doi: 10.1177/13623613211019162 34348479

[40] Coolican J, Smith IM, Bryson SE. Brief parent training in pivotal response treatment for preschoolers with autism. Journal of Child Psychology and Psychiatry. 2010 Dec;51(12):1321–30. doi: 10.1111/j.1469-7610.2010.02326.x 21073457

[41] Gengoux GW, Abrams DA, Schuck R, Millan ME, Libove R, Ardel CM, et al. A pivotal response treatment package for children with autism spectrum disorder: An RCT. Pediatrics. 2019 Sep;144(3). doi: 10.1542/peds.2019-0178 31387868 PMC6856784

[42] Koegel RL, Koegel LK. Pivotal response treatments for autism: communication, social, & academic development. Baltimore: Paul H Brookes Publishing; 2006.

[43] Deniz EA. Çocuk merkezli oyun terapisinin otizmli çocuklarda sosyal becerilerin gelişimine etkisinin incelenmesi [Master’s dissertation]. Turkey: Necmettin Erbakan University; 2019.

[44] Hillman H. Child-centered play therapy as an intervention for children with autism: A literature review. International Journal of Play Therapy. 2018 Oct;27(4):198. 10.1037/pla0000083

[45] Salter K, Beamish W, Davies M. The effects of child-centered play therapy (CCPT) on the social and emotional growth of young Australian children with autism. International Journal of Play Therapy. 2016;25(2):78. https://psycnet.apa.org/doi/10.1037/pla0000012

[46] Kasari C, Gulsrud A, Paparella T, Hellemann G, Berry K. Randomized comparative efficacy study of parent-mediated interventions for toddlers with autism. Journal of Consulting and Clinical Psychology. 2015 Jun;83(3):554. doi: 10.1037/a0039080 25822242 PMC4755315

[47] Kossyvaki L, Papoudi D. A review of play interventions for children with autism at school. International Journal of Disability, Development and Education. 2016 Jan 2;63(1):45–63. 10.1080/1034912X.2015.1111303

[48] Odom SL, Strain PS. A comparison of peer‐initiation and teacher‐antecedent interventions for promoting reciprocal social interaction of autistic preschoolers. Journal of Applied Behavior Analysis. 1986 Mar;19(1):59–71. doi: 10.1901/jaba.1986.19-59 3710949 PMC1308041

[49] DiSalvo CA, Oswald DP. Peer-mediated interventions to increase the social interaction of children with autism: Consideration of peer expectancies. Focus on Autism and Other Developmental Disabilities. 2002 Nov;17(4):198–207. https://doi.org/10.1177%2F10883576020170040201

[50] Lawton K, Kasari C. Teacher-implemented joint attention intervention: pilot randomized controlled study for preschoolers with autism. Journal of Consulting and Clinical Psychology. 2012 Aug;80(4):687. doi: 10.1037/a0028506 22582764

[51] O’Keeffe C, McNally S. A systematic review of play-based interventions targeting the social communication skills of children with autism spectrum disorder in educational contexts. Review Journal of Autism and Developmental Disorders. 2021;1–31. 10.1007/s40489-021-00286-3

[52] Corbett BA, Swain DM, Coke C, Simon D, Newsom C, Houchins‐Juarez N, et al. Improvement in social deficits in autism spectrum disorders using a theatre‐based, peer‐mediated intervention. Autism Research. 2014 Feb;7(1):4–16. doi: 10.1002/aur.1341 24150989 PMC3943749

[53] Feuerstein R, Klein PS, Tannenbaum AJ, editors. Mediated learning experience (MLE): Theoretical, psychosocial and learning implications. London: Freund Publishing House Ltd.; 1991.

[54] Althoff CE, Dammann CP, Hope SJ, Ausderau KK. Parent-mediated interventions for children with autism spectrum disorder: A systematic review. The American Journal of Occupational Therapy. 2019 May;73(3):7303205010p1-3. doi: 10.5014/ajot.2019.030015 31120831

[55] Nevill RE, Lecavalier L, Stratis EA. Meta-analysis of parent-mediated interventions for young children with autism spectrum disorder. Autism. 2018 Feb;22(2):84–98. doi: 10.1177/1362361316677838 29490483

[56] Oono IP, Honey EJ, McConachie H. Parent‐mediated early intervention for young children with autism spectrum disorders (ASD). Evidence‐Based Child Health: A Cochrane Review Journal. 2013 Nov;8(6):2380–479. 10.1002/ebch.1952 PMC11831248 23633377

[57] Solomon R, Van Egeren LA, Mahoney G, Huber MS, Zimmerman P. PLAY project home consultation intervention program for young children with autism spectrum disorders: a randomized controlled trial. Journal of Developmental and Behavioral Pediatrics. 2014 Oct;35(8):475. doi: 10.1097/DBP.0000000000000096 25264862 PMC4181375

[58] Siller M, Hutman T, Sigman M. A parent-mediated intervention to increase responsive parental behaviors and child communication in children with ASD: A randomized clinical trial. Journal of Autism and Developmental Disorders. 2013 Mar;43(3):540–55. doi: 10.1007/s10803-012-1584-y 22825926 PMC3511916

[59] Pajareya K, Nopmaneejumruslers K. A pilot randomized controlled trial of DIR/Floortime™ parent training intervention for pre-school children with autistic spectrum disorders. Autism. 2011 Sep;15(5):563–77. doi: 10.1177/1362361310386502 21690083

[60] Shamseer L, Moher D, Clarke M, Ghersi D, Liberati A, Petticrew M, et al. Preferred reporting items for systematic review and meta-analysis protocols (PRISMA-P) 2015: elaboration and explanation. BMJ. 2015 Jan 2;349. doi: 10.1136/bmj.g7647 25555855

[61] Gibson JL, Pritchard E, de Lemos C. Play-based interventions to support social and communication development in autistic children aged 2–8 years: A scoping review. Autism & Developmental Language Impairments. 2021 Jun;6:23969415211015840. https://doi.org/10.1177%2F23969415211015840 10.1177/23969415211015840 PMC9620698 36381525

[62] Francis G, Deniz E, Torgerson C, Toseeb U. Play-based interventions for mental health: A systematic review and meta-analysis focused on children and adolescents with autism spectrum disorder and developmental language disorder. Autism & Developmental Language Impairments. 2022 Feb;7. 10.1177/23969415211073118 PMC9685160 36438159

[63] Higgins JPT, Thomas J, Chandler J, Cumpston M, Li T, Page MJ, et al. Cochrane handbook for systematic reviews of interventions. John Wiley & Sons; 2019. 10.1002/14651858.ED000142 PMC10284251 31643080

[64] Conrad CE, Rimestad ML, Rohde JF, Petersen BH, Korfitsen CB, Tarp S, et al. Parent-mediated interventions for children and adolescents with autism spectrum disorders: A systematic review and meta-analysis. Frontiers in Psychiatry. 2021:2067. doi: 10.3389/fpsyt.2021.773604 34867556 PMC8632873

[65] Kent C, Cordier R, Joosten A, Wilkes-Gillan S, Bundy A, Speyer R. A systematic review and meta-analysis of interventions to improve play skills in children with autism spectrum disorder. Review Journal of Autism and Developmental Disorders. 2020;7(1):91–118. 10.1007/s40489-019-00181-y

[66] Dijkstra-de Neijs L, Tisseur C, Kluwen LA, van Berckelaer-Onnes IA, Swaab H, Ester WA. Effectivity of play-based interventions in children with autism spectrum disorder and their parents: A systematic review. Journal of Autism and Developmental Disorders. 2021 Dec 2:1–30. doi: 10.1007/s10803-021-05357-2 34853960

[67] Lee JD, Meadan H. Parent-mediated interventions for children with ASD in low-resource settings: A scoping review. Review Journal of Autism and Developmental Disorders. 2021 Sep;8(3):285–98. 10.1007/s40489-020-00218-7

[68] Law ML, Singh J, Mastroianni M, Santosh P. Parent-Mediated Interventions for infants under 24 months at risk for autism spectrum disorder: A systematic review of randomized controlled trials. Journal of Autism and Developmental Disorders. 2021 Jul 8:1–22. doi: 10.1007/s10803-021-05148-9 34236590 PMC9114042

[69] Liu Q, Hsieh WY, Chen G. A systematic review and meta-analysis of parent-mediated intervention for children and adolescents with autism spectrum disorder in mainland China, Hong Kong, and Taiwan. Autism. 2020 Nov;24(8):1960–79. doi: 10.1177/1362361320943380 32720805

[70] Shalev RA, Lavine C, Di Martino A. A systematic review of the role of parent characteristics in parent-mediated interventions for children with autism spectrum disorder. Journal of Developmental and Physical Disabilities. 2020 Feb;32(1):1–21. 10.1007/s10882-018-9641-x

[71] Trembath D, Gurm M, Scheerer NE, Trevisan DA, Paynter J, Bohadana G, et al. Systematic review of factors that may influence the outcomes and generalizability of parent‐mediated interventions for young children with autism spectrum disorder. Autism Research. 2019 Sep;12(9):1304–21. doi: 10.1002/aur.2168 31294532

[72] Tarver J, Palmer M, Webb S, Scott S, Slonims V, Simonoff E, et al. Child and parent outcomes following parent interventions for child emotional and behavioral problems in autism spectrum disorders: A systematic review and meta-analysis. Autism. 2019 Oct;23(7):1630–44. doi: 10.1177/1362361319830042 30818965

[73] Harrop C. Evidence-based, parent-mediated interventions for young children with autism spectrum disorder: The case of restricted and repetitive behaviors. Autism. 2015 Aug;19(6):662–72. doi: 10.1177/1362361314545685 25186943

[74] Sterne JAC, Savović J, Page MJ, Elbers RG, Blencowe NS, Boutron I, et al. RoB 2: a revised tool for assessing risk of bias in randomised trials. BMJ 366: l4898. 2019. doi: 10.1136/bmj.l4898 31462531

[75] Sterne JA, Hernán MA, Reeves BC, Savović J, Berkman ND, Viswanathan M, et al. ROBINS-I: A tool for assessing risk of bias in non-randomised studies of interventions. BMJ. 2016 Oct 12;355. doi: 10.1136/bmj.i4919 27733354 PMC5062054

[76] Yogman M, Garner A, Hutchinson J, Hirsh-Pasek K, Golinkoff RM, Baum R, et al. The power of play: A pediatric role in enhancing development in young children. Pediatrics. 2018;142(3). doi: 10.1542/peds.2018-2058 30126932

[77] Ronald A, Hoekstra RA. Autism spectrum disorders and autistic traits: a decade of new twin studies. American Journal of Medical Genetics Part B: Neuropsychiatric Genetics. 2011;156(3):255–74. doi: 10.1002/ajmg.b.31159 21438136

[78] Rubenstein E, Chawla D. Broader autism phenotype in parents of children with autism: A systematic review of percentage estimates. Journal of Child and Family Studies. 2018;27(6):1705–20. doi: 10.1007/s10826-018-1026-3 29731598 PMC5933863

[79] Wainer AL, Hepburn S, McMahon Griffith E. Remembering parents in parent-mediated early intervention: An approach to examining impact on parents and families. Autism. 2017 Jan;21(1):5–17. doi: 10.1177/1362361315622411 26951325

