# Supplementary material for: Parent-mediated play‐based interventions to improve social communication and language skills of preschool autistic children: A systematic review and meta-analysis protocol
Source: PLoS One. 2022 Aug 15;17(8):e0270153. doi: 10.1371/journal.pone.0270153 (PMC9377609; doi:10.1371/journal.pone.0270153)
Supplement: S1 File — (DOC) [file pone.0270153.s001.doc]

**Supporting information**

**S1 Table. PRISMA-P checklist^1^**

| Section and topic | Item No | Checklist item | Check |
| --- | --- | --- | --- |
| ADMINISTRATIVE INFORMATION | | |  |
| Title: |  |  |  |
| Identification | 1a | Identify the report as a protocol of a systematic review | Yes |
| Update | 1b | If the protocol is for an update of a previous systematic review, identify as such | N/A |
| Registration | 2 | If registered, provide the name of the registry (such as PROSPERO) and registration number | Yes |
| Authors: |  |  |  |
| Contact | 3a | Provide name, institutional affiliation, e-mail address of all protocol authors; provide physical mailing address of corresponding author | Yes |
| Contributions | 3b | Describe contributions of protocol authors and identify the guarantor of the review | Yes |
| Amendments | 4 | If the protocol represents an amendment of a previously completed or published protocol, identify as such and list changes; otherwise, state plan for documenting important protocol amendments | N/A |
| Support: |  |  |  |
| Sources | 5a | Indicate sources of financial or other support for the review | Yes |
| Sponsor | 5b | Provide name for the review funder and/or sponsor | Yes |
| Role of sponsor or funder | 5c | Describe roles of funder(s), sponsor(s), and/or institution(s), if any, in developing the protocol | Yes |
| INTRODUCTION | | |  |
| Rationale | 6 | Describe the rationale for the review in the context of what is already known | Yes |
| Objectives | 7 | Provide an explicit statement of the question(s) the review will address with reference to participants, interventions, comparators, and outcomes (PICO) | Yes |
| **METHODS** |  |  |  |
| Eligibility criteria | 8 | Specify the study characteristics (such as PICO, study design, setting, time frame) and report characteristics (such as years considered, language, publication status) to be used as criteria for eligibility for the review | Yes |
| Information sources | 9 | Describe all intended information sources (such as electronic databases, contact with study authors, trial registers or other grey literature sources) with planned dates of coverage | Yes |
| Search strategy | 10 | Present draft of search strategy to be used for at least one electronic database, including planned limits, such that it could be repeated | Yes |
| Study records: |  |  |  |
| Data management | 11a | Describe the mechanism(s) that will be used to manage records and data throughout the review | Yes |
| Selection process | 11b | State the process that will be used for selecting studies (such as two independent reviewers) through each phase of the review (that is, screening, eligibility and inclusion in meta-analysis) | Yes |
| Data collection process | 11c | Describe planned method of extracting data from reports (such as piloting forms, done independently, in duplicate), any processes for obtaining and confirming data from investigators | Yes |
| Data items | 12 | List and define all variables for which data will be sought (such as PICO items, funding sources), any pre-planned data assumptions and simplifications | Yes |
| Outcomes and prioritization | 13 | List and define all outcomes for which data will be sought, including prioritization of main and additional outcomes, with rationale | Yes |
| Risk of bias in individual studies | 14 | Describe anticipated methods for assessing risk of bias of individual studies, including whether this will be done at the outcome or study level, or both; state how this information will be used in data synthesis | Yes |
| Data synthesis | 15a | Describe criteria under which study data will be quantitatively synthesised | Yes |
|  | 15b | If data are appropriate for quantitative synthesis, describe planned summary measures, methods of handling data and methods of combining data from studies, including any planned exploration of consistency (such as I^2^, Kendall’s τ) | Yes |
|  | 15c | Describe any proposed additional analyses (such as sensitivity or subgroup analyses, meta-regression) | Yes |
|  | 15d | If quantitative synthesis is not appropriate, describe the type of summary planned | N/A |
| Meta-bias(es) | 16 | Specify any planned assessment of meta-bias(es) (such as publication bias across studies, selective reporting within studies) | Yes |
| Confidence in cumulative evidence | 17 | Describe how the strength of the body of evidence will be assessed (such as GRADE) | Yes |

*Note.* 1= Shamseer et al. [60].

**S2 Table. Search Strings ¹**

| Search Strings | Database (e.g., Ebscohost) | | |
| --- | --- | --- | --- |
|  | Search Dates | Number of results | Number of Studies Downloaded |
| Play (Title) AND “language” (Full text) AND PY=(2000-2021) |  |  |  |
| Play (Title) AND "language delay" (Full text) AND PY=(2000-2021) |  |  |  |
| Play (Title) AND "language disorder" (Full text) AND PY=(2000-2021) |  |  |  |
| Play (Title) AND "speech" (Full text) AND PY=(2000-2021) |  |  |  |
| Play (Title) AND "communication" (Full text) AND PY=(2000-2021) |  |  |  |
| Play (Title) AND "specific language impairment" (Full text) AND PY=(2000-20211) |  |  |  |
| Play (Title) AND "SLI" (Full text) AND PY=(2000-2021) |  |  |  |
| Play (Title) AND "Slow talk" (Full text) AND PY=(2000-2021) |  |  |  |
| Play (Title) AND "developmental language disorder" (Abstract) AND PY=(2000-2021) |  |  |  |
| Play (Title) AND "autis*" (Title) AND PY=(2000-2021) |  |  |  |
| Play (Title) AND pervasive* (Title) AND PY=(2000-2021) |  |  |  |
| Play (Title) AND PDP (Title) AND PY=(2000-2021) |  |  |  |
| Play (Title) AND Asperger (Title) AND PY=(2000-2021) |  |  |  |
| JASPER (Joint Attention, Symbolic Play, Engagement & Regulation) (Title) & "autis*" (Full text) AND PY=(2000-2021) |  |  |  |
| ASAP (Advancing Social-communication And Play) (Title) AND PY=(2000-2021) |  |  |  |
| DIR "Developmental Individual-difference Relationship-based model" (Title) AND PY=(2000-2021) |  |  |  |
| CCPT (Child-Centered Play Therapy) (Title) AND “autis*” (Full text) AND PY=(2000-2021) |  |  |  |
| Filial therapy (Title) AND PY=(2000-2021) |  |  |  |
| Lego therapy (Title) AND PY=(2000-2021) |  |  |  |
| Canine-assisted play (Title) AND PY=(2000-2021) |  |  |  |
| Theraplay (Title) AND PY=(2000-2021) |  |  |  |
| Jungian Play (Title) AND PY=(2000-2021) |  |  |  |
| Pivotal Response Treatment (PRT) (Title) AND PY=(2000-2021) |  |  |  |

*Note.* 1= Francis et al. [62].

**S3 Table. Data Extraction Form**

| Study | | | | | | Sample | | | | | | | Study Design | | | | | | |
| --- | --- | --- | --- | --- | --- | --- | --- | --- | --- | --- | --- | --- | --- | --- | --- | --- | --- | --- | --- |
| Study ID | Year | Country | Author | Title | Publication type | Target group  (child / parent) | Sample characteristics (type of SLCNs) | Previous clinical diagnosis of autism | Autism screening measure | Age | Gender ratio | Sample Size | Experimental design | Control group | Control treatment | Predictor variable | Outcome  variable/s | Measures | Measures standardised |
|  |  |  |  |  |  |  |  |  |  |  |  |  |  |  |  |  |  |  |  |

**S3 Table. Data Extraction Form (continued)**

| Intervention | | | | | | | | | | | | |
| --- | --- | --- | --- | --- | --- | --- | --- | --- | --- | --- | --- | --- |
|
| Intervention name | Intervention validated? | Type of play | Intervention setting | Intervention implementer | Intervention mediator | Mediator trained? | Mediator training | Duration of mediator training | Parental involvement | Intervention length | Intervention frequency | Total intervention time  (hours) |
|  |  |  |  |  |  |  |  |  |  |  |  |  |

**S4 Table. Quantified outcomes**

| Descriptive Information | | | | Study Outcome | | Results | | | | | | | | | | | | | | |
| --- | --- | --- | --- | --- | --- | --- | --- | --- | --- | --- | --- | --- | --- | --- | --- | --- | --- | --- | --- | --- |
|  |  |  |  |  |  | Experimental Group | | | | Control Group | | | | Reported effects | | | Standardised mean Differences | | | |
| Study ID | Year | Author | Title | Outcome type | Outcome variables | Sample size | Mean | SD | SE | Sample size | Mean | SD | SE | Effect size | P | Effect size type | SMD  (Cohen’s d) | SE | P | CI |
|  |  |  |  |  |  |  |  |  |  |  |  |  |  |  |  |  |  |  |  |  |

**S5 Table. Risk-of-Bias Tool^1^**

| **Domain 1: Risk of bias arising from the randomisation process or confounding effect** | | |
| --- | --- | --- |
| **Questions** | **Comments** | **Response** |
| **RCT Designs** |  |  |
| 1.1 Was the allocation sequence random? |  | Y / PY / PN / N / NI |
| 1.2 Was the allocation sequence concealed until participants were enrolled and assigned to interventions? |  | Y / PY / PN / N / NI |
| **QED Designs** |  |  |
| 1.3 Is there potential for confounding of the effect of intervention in this study? e.g. allocations using baseline equivalents, appropriate statistical analysis of difference on pre-tests, etc. |  | Y / PY / PN / N / NI |
| 1.4. Was selection of participants into the study (or into the analysis) based on participant characteristics observed after the start of intervention? |  | Y / PY / PN / N / NI |
| **Risk-of-bias judgement** |  | Low / Some concerns/ High |
| **Optional:** What is the overall predicted direction of bias for this outcome? |  | NA / Favours experimental / Favours comparator / Towards null /Away from null / Unpredictable |
| **Domain 2: Risk of bias due to deviations from the intended interventions** | | |
| **RCT Designs** | | |
| 2.1. Were participants aware of their assigned intervention during the trial? |  | Y / PY / PN / N / NI |
| 2.2. Were carers and people delivering the interventions aware of participants' assigned intervention during the trial? |  | Y / PY / PN / N / NI |
| **QED Designs** |  |  |
| 2.3Was the intervention clearly defined |  | Y / PY / PN / N / NI |
| 2.4 Was the information used to define intervention groups  recorded at the start of the intervention? |  | Y / PY / PN / N / NI |
| **Risk-of-bias judgement** |  | Low / Some concerns/ High |
| **Optional:** What is the overall predicted direction of bias for this outcome? |  | NA / Favours experimental / Favours comparator / Towards null /Away from null / Unpredictable |
| **Domain 3: Missing outcome data** | | |
| 3.1 Were data for this outcome available for all, or nearly all, participants randomised? |  | Y / PY / PN / N / NI |
| 3.2 Is there evidence that the result was not biased by missing outcome data? |  | Y / PY / PN / N / NI |
| **Risk-of-bias judgement** |  | Low / Some concerns/ High |
| **Optional:** What is the overall predicted direction of bias for this outcome? |  | NA / Favours experimental / Favours comparator / Towards null /Away from null / Unpredictable |
| **Domain 4: Risk of bias in measurement of the outcome** | | |
| 4.1 Was the method of measuring the outcome inappropriate? |  | Y / PY / PN / N / NI |
| **Risk-of-bias judgement** |  | Low / Some concerns/ High |
| **Optional:** What is the overall predicted direction of bias for this outcome? |  | NA / Favours experimental / Favours comparator / Towards null /Away from null / Unpredictable |
| **Domain 5: Risk of bias in selection of the reported result** | | |
| 5.1 Were the data that produced this result analysed in accordance with a pre-specified analysis plan that was finalized before unblinded outcome data were available for analysis? |  | Y / PY / PN / N / NI |
| 5.2 Is the numerical result being assessed likely to have been selected, on the basis of the results, from... |  | Y / PY / PN / N / NI |
| 5.3 Did they have a priori protocol? |  | Y / PY / PN / N / NI |
| **Risk-of-bias judgement** |  | Low / Some concerns/ High |
| **Optional:** What is the overall predicted direction of bias for this outcome? |  | NA / Favours experimental / Favours comparator / Towards null /Away from null / Unpredictable |
| **Overall Risk of Bias Judgement** |  | Low / Some concerns/ High |

*Note.* 1= Francis et al. [62]. Y= Yes, PY= Probably Yes, PN= Probably No, N= No, NI=No

information
