# Supplementary material for: Parent-mediated play‐based interventions to improve social communication and language skills of preschool autistic children: A systematic review and meta-analysis protocol
Source: PLoS One. 2022 Aug 15;17(8):e0270153. doi: 10.1371/journal.pone.0270153 (PMC9377609; doi:10.1371/journal.pone.0270153)
Supplement: Attachment — Submitted filename: 6- Response to Reviewers.docx.docx [file pone.0270153.s003.docx]

**Table Summarising Response to Reviewers’ Comments**

| **Reviewer’s comments** | **Authors’ Responses** |
| --- | --- |
| **Reviewer #1:** I would like to thank the authors for their careful attention to the edits. I am very satisfied with these edits and only have one small question remaining, which pertains to the inclusion/exclusion. The authors have stated that children who were not screened for autism will be excluded but there is no mention of positive screening in the inclusion criteria. Therefore, it is not clear to me whether children screened as "high likelihood of autism" but not yet diagnosed will be included. If they are, the inclusion sentence could read as follows**: "Studies with a sample of autistic children, children who screened as "high likelihood for autism" on a validated screening too, or children with a previously diagnosed form of autism (e.g., Asperger’s syndrome, PDD-NOS, autistic spectrum disorder).** If they will be excluded, then the mention of screening in the exclusion criteria can be removed. | We appreciate the reviewer’s positive comments and are thankful for their very valuable contribution to our manuscript.  **Autism Diagnosis**  We agree with the reviewer’s point in the sense that the mentioned inclusion criteria should have been further clarified. To address this comment, we revised our inclusion criteria as follows:  Inclusion criteria   - *Studies with a sample of autistic children, including the ones who screened as "high likelihood for autism" on a validated autism screening measure, or children with a previously diagnosed form of autism (e.g., Asperger’s syndrome, PDD-NOS, autistic spectrum disorder).*   *See page 10, inclusion criteria 2* |
| **Reviewer #2:** I appreciate the authors had provided more explanations and justifications on the rationale of parent-mediated intervention and why it is important. | We appreciate the reviewer’s positive comments and are thankful for their contribution to our manuscript. |

**Additional Revisions:** The reference list has been checked for typing errors and all typing errors have been corrected. In addition, the following reference has been corrected: 6. Rubin KH, Fein GG, Vandenberg B. Play In PH Mussen & EM Hetherington (Eds.), Handbook of child psychology vol. 4: socialization, personality, and social development (Vol. 4, pp. 693-774). New York; Chichester: Wiley, 1983.
